# UK media reporting of NICE recommendation of crizanlizumab for patients with sickle cell disease

**DOI:** 10.1002/jha2.623

**Published:** 2022-12-25

**Authors:** Richard J Buka, Noémi Roy, Phillip LR Nicolson

**Affiliations:** ^1^ Institute of Cardiovascular Sciences University of Birmingham Birmingham UK; ^2^ HaemSTAR Birmingham UK; ^3^ Department of Haematology Oxford University Hospitals NHS Foundation Trust Oxford UK

**Keywords:** Crizanlizumab, Media, Sickle cell disease

## Abstract

Crizanlizumab was recommended for use in patients with sickle cell disease in the UK in October 2021 and received widespread media coverage. Accuracy of reporting is paramount in building trust with this group of patients who are often wary of the medical profession. We carried out an analysis of internet‐based news articles and applied a validated scoring system to assess quality. 21 articles from 19 media organisations were identified. 71% of articles stated unproven benefits of the drug and only 14% were of satisfactory quality. The former was largely due to quoting of two NHS England press releases. Overstating of drug efficacy may be detrimental to the need to address healthcare inequalities.

1

Sickle cell disease is the most prevalent inherited disease in the United Kingdom (UK) and affects about 15,000 people.[1] In October 2021, the National Institute for Health and Care Excellence (NICE) recommended that crizanlizumab, a monoclonal antibody directed against P‐selectin, be offered to patients in the UK as part of a managed access agreement.[2] This was based on the results of a phase II trial (SUSTAIN) where 198 patients were randomised to low‐dose or high‐dose crizanlizumab, or placebo and followed‐up for one year. The study met its primary endpoint showing reduced sickle‐cell related pain crises in the high‐dose group compared with placebo but there was no improvement in quality of life and impact on survival was not investigated.[3]

Awareness of sickle cell disease is limited among healthcare staff and poor outcomes have been reported to be secondary to poor care and negative attitudes often underpinned by racism.[4] Distrust of the medical profession has been identified as a reason for poor uptake of health interventions in black and ethnic minority individuals, the majority of people living with sickle cell disease.[5] As crizanlizumab is the first new agent for sickle cell disease approved for use in the UK in the last two decades, the news received widespread media attention, much of which has been overtly positive. For trust to be fostered between this patient group and the medical profession, accurate and honest reporting of the benefits and drawbacks of therapies are important. As the lay press is an important source of information for patients and the public, and healthcare articles in general have previously been reported to be of variable quality,[6] we aimed to assess the quality and accuracy of the media coverage of the approval and first usage of crizanlizumab.

On 24^th^ May 2022, we searched the websites of 25 of the largest UK national and regional news organisations for articles containing the word ‘crizanlizumab’ from 1^st^ October 2021 until 28^th^ February 2022, the time between NICE recommendation and first use. These were all general news websites, not health‐related sites. 7 were regional newspapers (e.g. Evening Standard), 5 national tabloid newspapers (e.g. The Daily Express), 3 national broadsheet newspapers (e.g. The Times), 3 national broadcasters (e.g. BBC) and 2 online‐only news sites (e.g. HuffPost). A full list of organisations with descriptions is given in supplementary table 2.

We collected data on an array of metrics including those required to perform quality scoring according to the method of Robinson et al [6] which we amended slightly. This tool scores articles based on scores of ‐1, 0, or 1 across 20 domains. Domains were scored in three ways: seven were either ‐1, 0, or 1, nine were 1 or 0, and four were 0 or 1. The score for each domain is added to give a total article quality score (supplementary table 1). Three co‐authors independently scored the articles and where there were differences, these were discussed and a consensus was reached. We analysed each article's full text to identify themes which were derived as they emerged from the data on a sentence‐by‐sentence basis. Data was recorded in Microsoft 356 Excel (Microsoft Corporation, Redmond, WA) and figures constructed using Prism 9.0 (GraphPad Software, La Jolla, CA).

Of 25 websites searched, we identified 21 articles from 19 organisations (supplementary table 1). The median length of articles was 670 words (range 261–1168) and the contents of articles are summarised in table 1. Notably no articles discussed safety or toxicity of crizanlizumab and only 2 (9.5%) accurately reported the primary outcome of the SUSTAIN study: incidence of acute painful crises as an absolute risk. Only 2 (9.6%) articles explained the nature of the managed access scheme meaning that the drug is initially only available to approximately 300 patients.

Article quality scores are shown in figure 1 and the mean total quality score was ‐3.5 (SD 2.4). 18 (85.7%) articles were unsatisfactory using a pre‐specified cut‐off of ≥0 as determined by Robinson et al.[6] The best article was from the British Broadcasting Corporation (BBC) with a score of 1. Article length was not correlated with quality score (data not shown).

**FIGURE 1:**
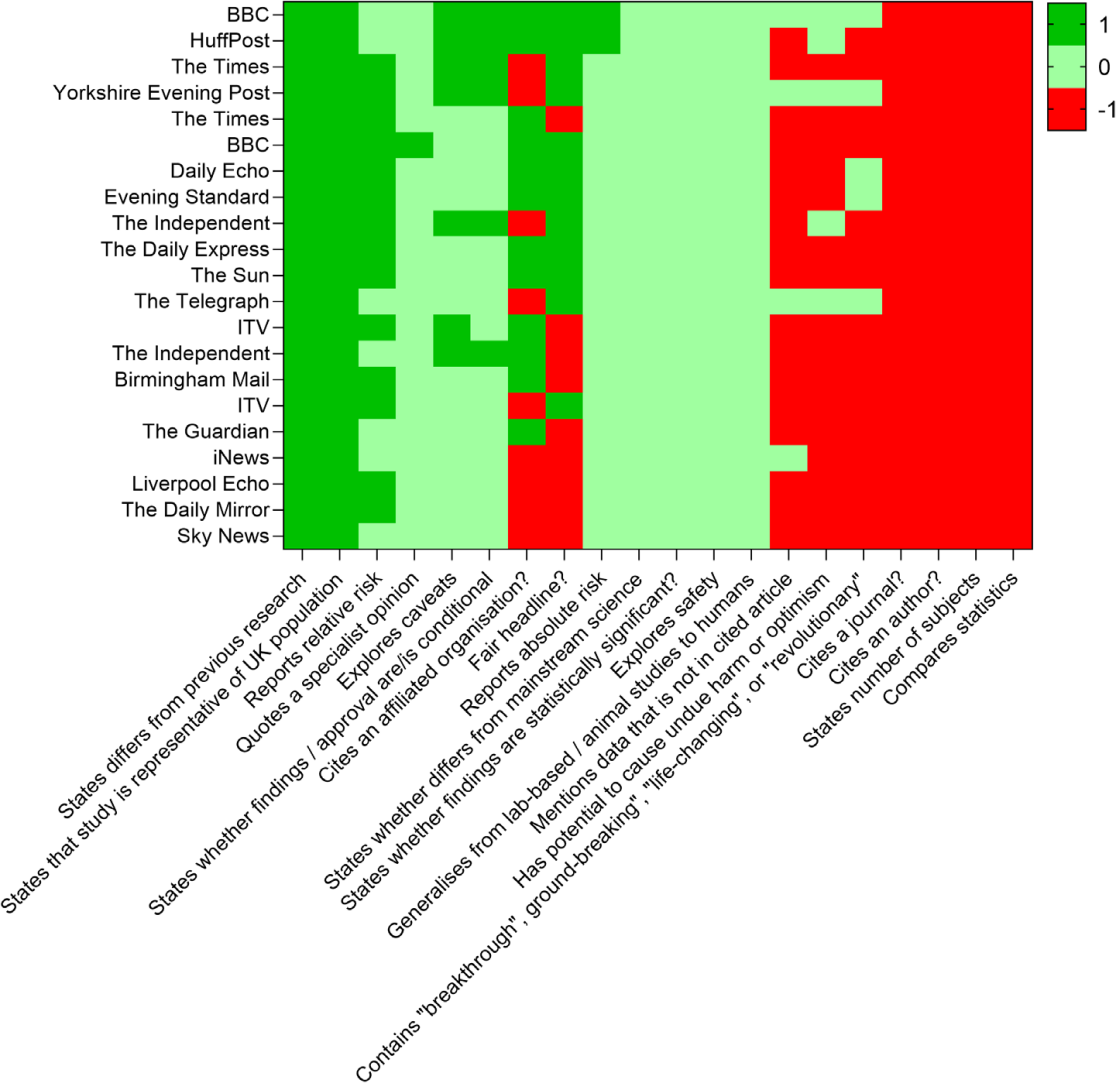
Heatmap showing scores across all domains for each article. The x‐axis shows each individual quality point (domain). Domains were scored in three ways: seven were either ‐1, 0, or 1, nine were 1 or 0, and four were 0 or 1. Scores of 1 are symbolised by a dark green square, 0 by a light green square, and ‐1 by a red square. Publishing organisations are listed on the y‐axis and ordered by total score (top to bottom: best to worst)

The majority of studies reported benefits of crizanlizumab that were not evident in the trial such as improvement in survival in 13 (61.9%), and improved quality of life in 16 (76.2%). Only 5 (23.8%) articles discussed limitations of the study and the subsequent NICE recommendation. Superlative adjectives to describe crizanlizumab were widely used both in headlines in 8 (38.1%) and in the main text in 18 (85.1%).

Many of the inaccurate claims were due to the quoting of two healthcare leaders in two NHS England press releases: [7, 8]

“*This* *is* *a historic* *moment for peo* *ple with sickle cell* *disease who will be given* *their first new treatment in over two decades* *. This revolutionary treatment* *will help to save lives*, *allow patients to have a better quality of life* *and reduce trips to A&E by almost half.”*

*
“It's fantastic that our first NHS patients* *have been given this ground‐breaking and historic new treatment for sickle cell disease – the first in over two decades*. *This revolutionary treatment will allow patients to have a better quality of life, reduce trips to A&E by almost half and ultimately help to save lives.”*

In general, patients’ and charities’ reactions to the news was positive:

*“A new treatment brings hope and might make dreaming possible again.”*

*“Seeing developments in treatment is extremely reassuring.”*

*“We are extremely excited and relieved that finally a new treatment has been developed.”*

but this was often caveated by concerns about access to treatment and frustration about the general lack of, or slow, progress

*“I wonder how doctors will make the decision of who to give it to given that so many people have the condition?”*

*“It's a shame that it has taken 20 years; I guess good things take time.”*

Other themes included widespread reporting of sickle cell disease as a severe disease, patient experiences, stigma, and patients with sickle cell disease being underserved.

To our knowledge, this is the first study to analyse UK media coverage of a news story about sickle cell disease. We have found that articles were generally of poor quality and we have identified pertinent themes (See Table 1).

**TABLE 1:** Summary of contents of articles

Criterion	n	%
Explained nature of sickle cell disease	21	100
Explained that sickle cell disease mostly affects black people	21	100
Explored health inequalities	13	61.9
Explored meaning of managed access approval	2	9.6
Reported drug mechanism	18	85.7
Discussed drug safety	0	0
Reported absolute risk	3	14.2
Reported relative risk	16	76.2
Stated drug reduces emergency department attendances	16	76.2
Discussed limitations of SUSTAIN trial and/or NICE recommendations	5	23.8
Stated benefits not in SUSTAIN trial	15	71.4
*Stated drug improves survival*	13	61.9
*Stated drug improves quality of life*	16	76.2
Used a superlative	18	85.7
*Revolutionary*	15	71.4
*Life‐changing*	10	47.6
*Ground‐breaking*	5	23.8
*Breakthrough*	1	4.8
*Used a superlative in headline*	8	38.1
Quotes		
*Quoted a patient*	16	76.2
*Quoted Sickle Cell Society*	14	66.7
*Quoted NHS England Chief Executive*	12	57.1
*Quoted NHS England and Improvement Director of Health Inequalities*	8	38.1
*Quoted a haematologist*	1	4.8

In the SUSTAIN trial, high‐dose crizanlizumab reduced annual episodes of painful crises requiring admission from on average 2.98 in the placebo group to 1.63 in the treatment group.[3] Importantly, high‐dose treatment did not improve quality of life scores and the trial did not evaluate survival. Indeed, the NICE Technology Appraisal Guidance thoroughly discusses the limitations of the evidence; that the SUSTAIN trial was small, of short duration, and did not report on survival, acute chest syndrome, or incidence of painful crises that were managed at home.[2] Due to the general lack of explanation or critique of the limitations and misreporting of statistics, 16 (76.1%) articles were deemed to be at risk of causing undue optimism which may provide unrealistic hope which, if not realised, may diminish trust between patients, families, and the medical profession. This could take the form of patients’ conditions not being alleviated to the degree suggested in the media coverage, despite the scientific reports being clear of the new drug's limited efficacy. Alternatively, the incorrect reporting of a new therapy as ‘groundbreaking’ when there is insufficient evidence of its long term benefits could hamper further efforts to dedicate adequate funding to gather the additional evidence if the general perception is that sufficient progress has been made in this field. This was compounded by the liberal use of superlatives, something which has been noted before in the media.[9] Using their scoring system we found that the mean quality score of articles was much lower than in Robinson et al's broader study.[6] In their analysis of 160 newspaper articles across 8 national daily newspapers, the mean score was 5.53 with 89% ≥0 and 13% > 10 whereas in our study the average score was ‐2.95 (SD 1.91) with only 14% ≥0 and none > 10. No citation of original research, superficial or misleading reporting of study outcomes, failure to explore caveats, and use of superlatives were the principal reasons for which studies scored badly.

Despite our criticisms, there were a number of things that articles did well including explanations of sickle cell disease, communication of its severity using patient testimony, and mentioning health inequity. Furthermore, many articles were heavily dependent on NHS England press releases,[7,8] which, to a non‐specialist journalist, should be regarded as a reliable source, and it is from these press releases that many of the spurious statements about drug efficacy stem. Articles did well to include patients’ opinions and patient reaction to the news was largely positive. However, strong themes were the disappointment that this new treatment has taken so long to be developed, and concerns over accessibility to treatment.

There are limitations to this study. Firstly, for practical reasons, we have only studied written internet articles from major news organisations. Whilst 65% of the public use the internet for their news, television is used by 75% and social media by 45%. Only 35% use print media but the latter is likely to be very similar to the internet‐based articles from the same organisations.[10] It is also not possible to assess the direct impact on the views of patients and their relatives. Limited data exists on the consumption of media in the UK in the black population, the population from which the majority of patients with sickle cell disease come. The pattern of news consumption is different in minority ethnic groups [10] but our broad cross‐section of news sources is likely to have captured at least some of the sites used by this population. We also slightly adapted the previously validated scoring system of Robinson et al. but our amendments were minimal and it is still reasonable to draw valid comparisons with our data and the data in their study.[6] Our study is strengthened by the utilisation of three independent scorers.

UK media coverage of the approval and first use of crizanlizumab was of poor quality and over‐optimistic, fuelled by superlative‐filled, misleading statements made by healthcare leaders. This can be seen within the wider context of problematic media reporting of health stories which can have significant public health consequences.[11] This is on a background of evidence of poor care for patients with sickle cell disease caused by low levels of awareness and insufficient training, as well as prejudicial attitudes, lack of respect, and patients being disbelieved, often underpinned by racism.[4] NICE recognise that there is an unmet need for effective treatments for people with sickle cell disease who face health inequalities.[2] Healthcare leaders overplaying the benefits of crizanlizumab may be in part an attempt to address this but it is at best questionable whether this is beneficial. It is certainly plausible that overstating progress could signal to the public that the problem is solved which may divert resources away from this patient group. Further research is required in this area. Finally, healthcare leaders have a duty to speak the truth and communicate research findings in an honest way.

## AUTHOR CONTRIBUTIONS

RB designed the study, collected data, and wrote the manuscript. NR and PN collected data and edited the manuscript.

## FUNDING INFORMATION

This work required no external funding.

## CONFLICT OF INTEREST

RB has received a research grant from AstraZeneca. NR has no conflicts of interest to disclose. PN has received research grants from Novartis, Principia, Rigel, and Sanofi and speaker fees from Bayer, Sobi, Takeda and Grifols.

## ETHICAL APPROVAL STATEMENT

As this research was carried out using publicly accessible sources, ethical approval was not required.

## Supporting information

Supporting Information

Supporting Information

## Data Availability

The data that support the findings of this study are available from the corresponding author upon reasonable request.
