# Supplementary material for: UK media reporting of NICE recommendation of crizanlizumab for patients with sickle cell disease
Source: EJHaem. 2022 Dec 25;4(1):13–7. doi: 10.1002/jha2.623 (PMC9928781; doi:10.1002/jha2.623)
Supplement: Supplementary file 1 — Supporting Information [file JHA2-4-13-s001.docx]

**Supplementary table 1.** Scoring system for article quality

| Score 1 or -1 |
| --- |
| Cites a journal? |
| Cites an author? |
| Cites an affiliated organisation? (e.g. Sickle Cell Society) |
| States number of subjects in trial |
| Explores how this study differs from previous research |
| Compares statistics (1). Are statistics misused or misrepresented? (-1) |
| Is the headline a fair reflection of the article and evidence? |
| Score 1 or 0 |
| States whether the findings (and/or drug approval) are preliminary or conclusive |
| States whether differs from mainstream science |
| States whether findings are statistically significant? |
| Reports absolute risk |
| Reports relative risk |
| Explores safety of the intervention |
| Explores caveats to the conclusions of the study or the efficacy of the treatment |
| Quotes a specialist opinion (haematologist) |
| Article is clear that the treatment is for patients with sickle cell disease |
| Score 0 or -1 |
| Mentions data or findings that are not reported in the study (e.g. prolongs survival or improves quality of life) |
| Has potential to cause undue harm or optimism |
| Generalises from lab-based / animal studies to humans without explicitly saying so (not applicable, so score 0) |
| Uses any of the following to describe the drug: “life-changing”, “breakthrough”, “ground-breaking”, or “revolutionary” |

**Supplementary table 2.** News organisations and article headlines

| News organisation | Description of organisation | Headline | Fair headline? |
| --- | --- | --- | --- |
| BBC | Publicly funded national broadcaster, print media online only | First New Treatment for Sickle Cell Disease in 20 Years" | Yes |
| BBC | As above | New sickle cell treatment given to first patients in England | Yes |
| Birmingham Mail | Regional newspaper, Birmingham, print and online | Walsall woman given new lease of life with groundbreaking drug to battle chronic ‘stabbing pain’ It is the first time a treatment for the disease has become available on the NHS in 20 years | No |
| Chronicle Live | Regional newspaper, Newcastle upon Tyne, print and online | NA |  |
| Daily Echo | Regional newspaper, Southampton, print and online | NHS approve first sickle cell disorder treatment in 20 years - how it works | Yes |
| Evening Standard | Regional newspaper, London, print and online | Headline: New hope for sickle cell disease as first drug in 20 years approved | Yes |
| HuffPost | News website, online only | Here’s What We Know About The New Drug For Sickle Cell Disease. NHS chief hails ‘historic moment’ for drug that could reduce painful episodes and hospitalisation. | Yes |
| iNews | News website, online only | Sickle cell disease patients ‘given hope for the future’ with first new treatment in 20 years. ‘We’ve been neglected when it comes to new treatments, and we needed a win, and for me this is it’ | Yes |
| ITV | Public broadcaster, part public funding, part advertising, written media online only | Sickle cell disease: Mother welcomes ‘revolutionary’ new drug but why has it taken so long? | No |
| ITV | As above | Manchester teenager becomes the first in the North West to trial ‘life changing’ sickle cell drug | No |
| Liverpool Echo | Regional newspaper, Liverpool, print and online | NHS issues new ‘ground-breaking’ sickle cell treatment on first patients. Crizanlizumab, is the first new treatment for sickle cell disease in over two decades | No |
| Manchester Evening News | Regional newspaper, Manchester, print and online | NA |  |
| Metro | Free, national newspaper, print and online | NA |  |
| Sky News | Independent news broadcaster, written media online only | Sickle cell disease: Thousands to benefit from new life-changing drug treatment | No |
| The Daily Express | National tabloid newspaper, print and online | New treatment for sickle cell disease rolled out on NHS | Yes |
| The Daily Mail | National tabloid newspaper, print and online | NA |  |
| The Daily Mirror | National tabloid newspaper, print and online | ‘Groundbreaking’ new drug to combat agonising sickle cell disease rolled out on NHS | No |
| The Guardian | National broadsheet newspaper, print and online | NHS England to offer breakthrough treatment for sickle cell disease. Crizanlizumab is the first new therapy for 20 years for serious and lifelong health condition | No |
| The Independent | National broadsheet newspaper, online only since 2016 | New hope for sickle cell disease as first new drug in 20 years is approved. NHS England hopes new drug will help patients suffering from painful sickle cell crises | Yes |
| The Independent | As above | Sickle cell patients given new ‘life-saving’ treatment on the NHS, Crizanlizumab will reduce chronic pain and trips to A&E while improving patients’ quality of life | No |
| The Sun | National tabloid newspaper, print and online | GREEN LIGHT First new treatment for sickle cell disease for 20 years will be made available on NHS | Yes |
| The Telegraph | National broadsheet newspaper, print and online | The pain doesn’t even come close to pins and needles’: sickle cell sufferers hail new treatment | Yes |
| The Times | National broadsheet newspaper, print and online | Crizanlizumab, first new treatment in decades, gives sickle cell disease sufferers hope | Yes |
| The Times | As above | First sickle cell patients receive ‘life-changing’ drug | No |
| Yorkshire Evening Post | Regional newspaper, Yorkshire, print and online | Symptoms of sickle cell disease as NHS to offer first new treatment in 20 years | Yes |
